# A school-based intervention of screening a movie to increase hepatitis B vaccination levels among students in Uttar Pradesh, India: impact on knowledge, awareness, attitudes and vaccination levels

**DOI:** 10.1186/s41124-017-0027-y

**Published:** 2017-06-13

**Authors:** Gourdas Choudhuri, Rajesh Ojha, T. S. Negi, Varun Gupta, Shipra Saxena, Arundhati Choudhuri, Sanjoy Pal, Jui Choudhuri, Alok Sangam

**Affiliations:** 1 HOPE (Health Oriented Programs and Education) Initiative, www.hope.org.in, 422, Aradhana, Eldeco 2, Rae Bareli Road, Lucknow, UP 226025 India; 2 Department of Gastroenterology and Hepatobiliary Sciences, Fortis Memorial Research Institute, Sector 44, Gurgaon, Haryana 122002 India

**Keywords:** Hepatitis B virus, School program, Screening a movie, Attitude and practice, Vaccination, India

## Abstract

**Background:**

India is home to one in 14 of all chronic hepatitis B virus (HBV) cases, meaning that it is important to develop HBV interventions that are applicable in the Indian context. Vaccination is the foremost tool for interrupting the HBV infection cycle. HBV vaccination was not included in India’s government-sponsored expanded immunisation program until 2011, and many children born earlier remain unvaccinated. This study sought to observe the impact of the HOPE Initiative’s school-based intervention to increase vaccination coverage by increasing HBV awareness among students in Lucknow, Uttar Pradesh.

**Methods:**

At 430 schools in the administrative areas within and surrounding Lucknow, students viewed an educational documentary film on HBV and completed two questionnaires, one immediately before the screening and the other six weeks later. Both questionnaires asked the same 14 questions, which were organized into five domains: knowledge of the magnitude of the problem of HBV; knowledge of modes of HBV transmission; knowledge of consequences of HBV infection; awareness of HBV; and attitudes regarding HBV. The baseline questionnaire also asked students whether they had been vaccinated against HBV. At two-year follow-up, researchers measured vaccination levels at a subset of 30 intervention schools and six non-intervention schools to further assess the impact of the intervention.

**Results:**

Baseline questionnaires were completed by 11,250 students, and post-intervention questionnaires, by 9698 students. Scores for knowledge about the magnitude of the HBV problem improved from 41% at baseline to 74% at follow-up, and scores for knowledge about modes of transmission, from 38% to 75% (*p* < 0.05 for both). The baseline HBV vaccination level among students receiving the intervention was 21%. Two years after the intervention, 45% of students (*N* = 4284) reported being vaccinated at intervention schools compared to 22% (*N* = 1264) at non-intervention schools.

**Conclusions:**

The observed increases in HBV awareness, knowledge and vaccination levels in this study indicate that school-based interventions can be used to achieve higher vaccination coverage among Indian children. The documentary film was found to be an affordable tool for reaching large audiences. More studies are needed to validate the impact of this intervention and to explore its applicability to other social causes.

## Background

Hepatitis B virus (HBV) is a major public health problem, with almost 250 million people thought to be chronically infected worldwide [1, 2]. A safe, effective and affordable recombinant hepatitis B-DNA vaccine has become the foremost tool for interrupting the infection cycle. The vaccine has been available for three decades, and by 2013, was included in the national immunization programs of 183 World Health Organization (WHO) Member States [3]. Widespread vaccination has been shown to reduce the disease burden in several high-endemicity countries [4, 5].

A 2015 analysis of chronic HBV worldwide established India’s HBsAg prevalence at 1.46%. Although considerably lower than the estimated global HBsAg prevalence of 3.6%, India’s large population means the country is home to one in 14 of all chronic HBV cases [2]. Other research has found higher HBsAg prevalence for India, and better data are needed in order to describe the situation with greater certainty [6, 7].

The burden of disease from HBV has declined in many countries [8], mainly due to increased public awareness and effective, broadly targeted vaccination policies. India has lagged behind in both regards. Between 2007 and 2008, India introduced the HBV vaccine across ten of 22 states as part of a Universal Immunization Program [9]. The government’s universal HBV immunization program for infants did not target the entire country until 2011 [10, 11]. As a result, a large number of children born before 2011 in India, as well as almost all of the adult population, did not receive the HBV vaccine through the government-sponsored immunization program [12, 13]. Efforts to improve vaccination coverage for these unprotected children and adults have been undertaken primarily through awareness campaigns and social marketing activities carried out by nongovernmental organizations (NGOs) [14].

These measures have been designed with the understanding that HBV vaccination coverage in children greatly depends on caregivers’ awareness of the importance of having their children vaccinated. Some Indian children have opportunities to undergo vaccination either at private clinics or at free or subsidized “vaccination camps” for students, which are organized by school administrators in collaboration with NGOs. The reach of and response to these initiatives may vary in accordance with many factors, including the geographical setting and the socioeconomic background of the children [15].

In light of concerns over the need to ensure vaccination coverage for all children, the HOPE Initiative, a not-for-profit health promotion organization supported by technical assistance from WHO India, sought to increase HBV awareness among school children in and around Lucknow, Uttar Pradesh, in order to motivate them to get vaccinated. Lucknow is the capital of Uttar Pradesh and has poor health and social indices [16]. Around 2%–3% of the population in and around Lucknow is believed to be HBsAg positive [17, 18]. With a population of over 200 million, Uttar Pradesh is India’s most populous state [19]. It has the second-highest maternal mortality rate among Indian states and the poorest immunization practices [20].

The aim of the study was to observe the impact of the HOPE Initiative’s school-based program to increase vaccination coverage by increasing HBV awareness among student participants. We had tried several types of interventions earlier, such as distribution of pamphlets, debates, skits, but as our activities expanded in enrolling more members and geographical regions, we feel a documentary movie would be a standardized uniform means of intervention reducing dependance on school coordinators and their individual skills of communications.

In this study, we assessed awareness of HBV before and after student participants at 430 study schools screened an educational documentary film on HBV, with the expectation that increased awareness would motivate students to encourage their parents to vaccinate them against the virus, either in private clinics or through government-sponsored school-based vaccination programs. In addition, we explored the differences in HBV awareness and vaccination rates between schools of varying infrastructure levels. The study also compared student participant vaccination levels immediately before and two years after the documentary screening.

## Methods

### Selection of study schools

A consecutive sampling technique was used for study site selection in the administrative areas within and surrounding Lucknow, Uttar Pradesh. Sites were chosen from a list of all schools with secondary and higher secondary students (*N* = 2900) in the study area. From this initial list, 1500 schools matched the following three inclusion criteria: they enrolled students in classes nine through twelve, they were easily approachable to the study team, and they had suitable resources for the intervention. A letter inviting participation in the study was sent via mail to the principals of all potential study schools, and the study team waited one month for responses. At the end of the month, 436 schools in the study area had agreed to participate: 430 intervention schools, and six non-intervention schools were included for comparison.

### Study intervention

An educational intervention was administered as well as pre- and post-intervention assessment questionnaires. At the 430 study schools that agreed to serve as intervention schools, a documentary YouTube film entitled “Are you B safe?” was shown to students enrolled in the study. Depending on the language of instruction at each school, either the 18-min Hindi version or the 10-min English version of the film was screened for study participants [21]. The setting of the movie was urban and rural India, and it was made with specific attention to cultural appropriateness for the people of this region. Study schools screened the film three times annually between 2008 and 2014, with 250 to 300 student participants attending each screening.

### Study participants

Individual student participation in the study was voluntary. All secondary and higher secondary students (classes nine to twelve) attending selected study schools were eligible and were invited to participate. The initial step was to have the teacher send out a printed note to parents of interested student as well allow a member of HOPE team to explain to the students the purpose of the engagement. Students who obtained parental permission were invited to complete the pre-intervention questionnaire and to attend a documentary screening.

### Data collection

Participant student’s knowledge, attitudes and awareness about HBV were measured with self-administered, paper-based pre- and post-intervention assessment questionnaires. The pre-intervention assessment questionnaire was administered before the film was viewed and the post-intervention assessment questionnaire was administered six weeks after viewing the film. Both questionnaires asked the same 14 questions, which were organized into five domains: knowledge of the magnitude of the HBV problem (two questions); knowledge of modes of HBV transmission (five questions); knowledge of the consequences of HBV infection (three questions); awareness of HBV (two questions); and attitudes regarding HBV (two questions).

Participants were asked to answer all of the 14 yes/no questions. Responses were classified by the investigators as either “correct response” or “wrong response”. If the respondent had skipped any question or had written “I don’t know”, this was taken as “wrong response”. The percentages of correct answers to individual responses were noted.

The pre-intervention questionnaire contained two additional questions about participants’ HBV vaccination status. Respondents were asked if they had been vaccinated, and those who answered ‘no’ were asked to report reasons for not being vaccinated. The question about reasons was open-ended.

Three additional types of data collection took place for this study. First, researchers assessed how well-resourced each study school was by conducting an inventory of the school’s infrastructure and available facilities, using criteria defined for the purpose of this study (Table 1). Schools fulfilling eight to nine criteria were designated as “Category A” schools. Those fulfilling five to seven criteria were “Category B” schools, and those fulfilling less than five were “Category C” schools.

**Table 1:** Classification criteria for school infrastructure level

1. Well-maintained school building	
2. Well-equipped classrooms	
3. Science laboratory with adequate and functioning equipment	
4. Library with textbooks and reference materials	
5. Availability of playground equipment and facilities for sports	
6. Computer laboratory with adequate and functioning equipment, and regular electricity supply	
7. Auditorium or hall for activities	
8. Good drinking water facilities, including water cooler	
9. Toilets in good condition and handwashing facilities available	

Second, researchers determined which of the 430 intervention schools had previously held vaccination camps and which ones had not by asking the school administrators. Third, researchers assessed vaccination coverage two years after the documentary was screened by sampling students from 30 randomly selected intervention schools and from the six non-intervention schools. Permission was sought from school administrators to allow members of the HOPE team to ask students from classes nine to twelve if they had been vaccinated against HBV. Further, students were asked to take home a short form seeking the information from their parents and to bring the completed form back the following day.

Data collection took place from 2008 to 2014.

### Statistical analysis

This study was exploratory in nature, and formal sample size calculations were not considered necessary due to the unavailability of previous relevant research to inform such procedures. For comparing pre- and post-intervention findings, the chi-square test (with Yates’ correction) was performed using a 2 × 2 Table. A two-tailed *p*-value was taken. SPSS for Windows, Version 16.0, Chicago, SPSS, was used. For assessing the knowledge of students under the five broad domains, the total of correct responses to all questions under that domain was taken. For example the first domain had two questions. The first question was answered correctly by 48% (5400/11,250) of students and the second question was answered correctly by 34% (3825/11,250). The first domain thus had a total of 9225 (41%) correct responses out of a total of 22,500 responses.

### Research ethics

The study was approved by the HOPE Initiative Ethics Committee and reviewed on an annual basis to confirm continued ethical compliance. Study participation was voluntary and researchers first obtained formal permission from school principals to screen the movie and conduct the study in each school. Principals sent notes to the parents of students, describing study procedures and seeking permission for students to participate. Students who obtained written parental permission were invited to complete study procedures. These students were informed that they were free to refuse to participate in any part of the study or to withdraw at any time. For both the pre- and post-intervention questionnaires, students were asked to provide their names but were told that this information was optional.

Following the study, the Hope Initiative conducted vaccination campaigns in schools where campaigns had not previously taken place upon the request of school authorities. The names of students who underwent vaccination were recorded by HOPE team and school authorities for accounting purposes. Vaccination was provided only to students who had explicit written parental consent, and students were informed they could refuse.

## Results

### Study population

The baseline knowledge and attitudes survey elicited responses from 11,250 students aged [13–18] years studying in classes nine to twelve. Slightly less than half of students were male (*n* = 5433; 48%). Twenty percent of students attended Category A schools (highest level of infrastructure), while 41% attended Category B schools (middle level of infrastructure) and 39% attended Category C schools (lowest level of infrastructure).

Post-intervention surveys were completed by 9698 students (4850 males; 50%) six weeks after the students had viewed the documentary movie. In the post-intervention group, 21% of students were at Category A schools, 41% at Category B schools, and 38% at Category C schools. Around 1552 students(13.8%) were not available for post-intervention surveys due to school absences.

For the assessment of vaccination coverage two years after the intervention, 30 intervention schools were randomly selected and the vaccination status of 4284 students at those schools was determined. Researchers additionally determined the vaccination status of 1264 students at six non-intervention schools (Table 2).

**Table 2:** Assessment of vaccination coverage: schools and students in intervention and non-intervention groups at two-year follow-up

School Category	Number of Schools	Number of Students
Intervention schools		
Category A	6	1071
Category B	12	2411
Category C	12	802
Non-intervention schools		
Category A	1	122
Category B	3	340
Category C	2	802

### Student HBV knowledge, attitudes and vaccination status before the intervention

The baseline questionnaire administered to 11,250 students showed that overall knowledge about the magnitude of the HBV problem was 41% and that overall knowledge about modes of transmission was 38%. Sixty-two percent of students were aware that HBV causes jaundice but only 22% were aware of HBV causing liver cancer. It was also observed that only 33% of students were aware that HBV can be prevented by a vaccine and only 32% wanted HBV testing to be made free in their locality (Table 3).

**Table 3:** Detailed student responses to baseline and six-week post-intervention questionnaires

	Baseline (*n* = 11,250)				Follow-up (*n* = 9698)				*p*-value
	% correct, all schools	% correct, ‘A’ schools (*n* = 2242)	% correct, ‘B’ schools (*n* = 4612)	% correct, ‘C’ schools (*n* = 4396)	% correct, all schools	% correct, ‘A’ schools (*n* = 1996)	% correct, ‘B’ schools (*n* = 4022)	% correct, ‘C’ schools (*n* = 3680)	
Knowledge-related questions – magnitude of HBV problem	41%				74%				*p* < .05
1. Do you think hepatitis is a significant health problem in your community?	48%	58%	46%	45%	78%	82%	79%	75%	
2. Is hepatitis B common in the community?	34%	42%	31%	31%	70%	78%	72%	64%	
Knowledge-related questions – modes of transmission Does hepatitis B spread through:	38%				75%				*p* < .05
1. Transfusion of infected blood or re-use of disposable syringes	44%	48%	44%	42%	86%	89%	87%	86%	
2. From a healthy hepatitis B-carrier mother to her baby	38%	40%	39%	36%	75%	78%	74%	75%	
3. Sharing of razor blades/toothbrushes	35%	39%	34%	32%	77%	80%	76%	76%	
4. When an infected person coughs or sneezes	32%	38%	30%	29%	65%	70%	64%	64%	
5. Eating stale food	39%	45%	38%	35%	70%	73%	68%	67%	
Knowledge-related questions – consequences of infection	38%				65%				*p* < .05
1. Does hepatitis B cause jaundice?	62%	70%	64%	61%	90%	92%	92%	87%	
2. Does hepatitis B cause liver failure?	30%	32%	29%	30%	55%	60%	58%	50%	
3. Does hepatitis B cause liver cancer?	22%	22%	22%	22%	50%	52%	55%	44%	
Awareness-related questions	32%	52%	38%	32%	58%	82%	74%	55%	*p* < .05
1. Do you know that hepatitis B is preventable by vaccine?	33%	48%	44%	33%	64%	86%	79%	54%	
2. Do you know that HBV is treatable?	31%	56%	32%	32%	52%	78%	69%	56%	
Attitude-related question	34%	40%	37%	29%	66%	68%	66%	65%	*p* < .05
1. Do you want to be tested for HBV?	36%	38%	37%	34%	70%	73%	69%	70%	
2. Do you want free HBV testing in your locality?	32%	36%	35%	24%	62%	63%	63%	60%	
Are you vaccinated against HBV?	21%	53%	39%	7%					
If not, why not?									
1. Lack of awareness		14%	38%	48%					
2. Cost		13%	43%	43%					

For many questions, it was observed that students from Category A schools had higher proportions of correct responses than students from the other categories of schools. For example, more Category A students answered that HBV is a significant health problem (58%), and more were aware of the availability of a vaccine (48%) and of treatment for HBV (56%) than those from category B or C schools (Table 3).

Twenty-one percent of students reported that they had been vaccinated at baseline. Category A students had the highest vaccination rate (53%) followed by Category B (39%) and Category C (7%). Those not vaccinated cited lack of awareness (A: 14%, B: 38%, C: 48%) and cost (A: 13%, B: 43%, C: 43%) as the main barriers. Knowledge about modes of transmission was 75% in those vaccinated compared with 59% of those not vaccinated (data not shown). The same proportions of vaccinated students and unvaccinated students were found to be knowledgeable about consequences of the infection.

### Student HBV knowledge and attitudes six weeks after the intervention

Assessment of 9698 students at the six-week follow-up demonstrated knowledge increases in most of the five domains of questions. An improvement from 41% to 74% occurred in relation to knowledge about the magnitude of HBV as a public health problem and from 38% to 75% in relation to knowledge about modes of transmission. Seventy-eight percent of students now believed that HBV is a significant health problem in comparison to 48% before, and 86% were aware that transmission can occur via infected blood or used syringes in comparison to 44% before (Table 3).

When a category analysis was performed, it was observed that regardless of baseline knowledge a proportionate increase in knowledge was observed among all categories when questions were related to knowledge of the magnitude of the problem, modes of transmission and attitude. Almost 92% of students in categories A and B were aware that HBV causes jaundice after the intervention, while 87% of students in category C were aware. It was also observed that improvements in knowledge were greater in categories A and B in certain groups of questions. For example, the post-intervention improvement in knowledge that HBV is prevented by vaccine was greater in categories A and B (86% and 79% respectively) in comparison to Category C (54%). Overall, an improvement was observed in all categories post-intervention but it was substantially more in categories A and B than in category C in certain groups of questions (Table 3).

### Student HBV vaccination status at two-year follow-up

**Fig. 1:**
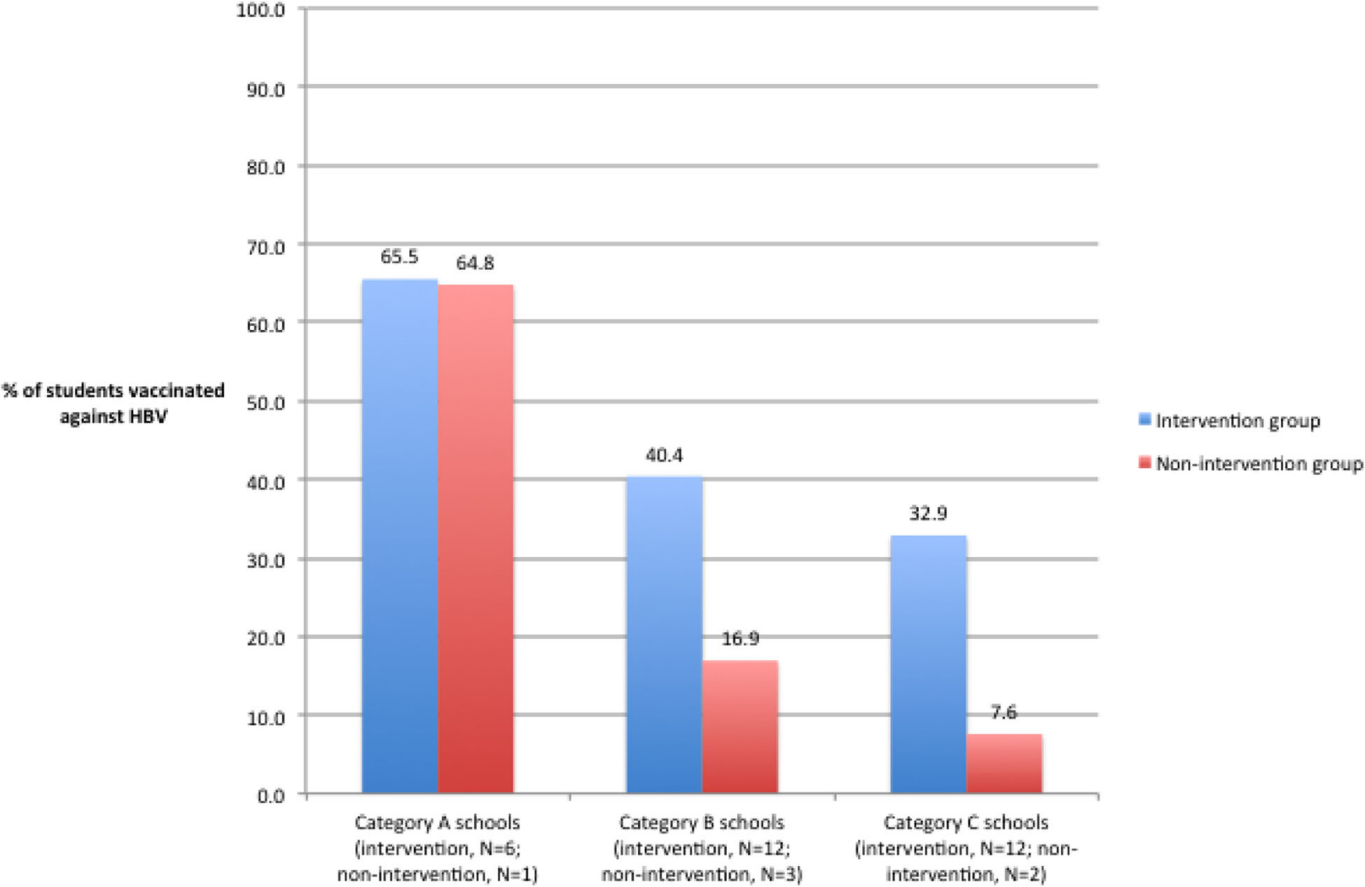
Post-intervention HBV vaccination levels at intervention schools (*N* = 30) and non-intervention schools (*N* = 6), by category of school infrastructure. The figure illustrates vaccination levels in all the three category of schools, post intervention of screening a video. It also illustrates vaccination level in all the three category of schools in which intervention was not done

Two years after the intervention, an HBV vaccine coverage level of 45% – more than double the baseline level of 21% – was observed in the 30 intervention schools that took part in this phase of the study. In the six non-intervention schools where vaccination levels were assessed, 22% of students were reported to be vaccinated. A comparison of intervention and non-intervention schools by category of infrastructure indicated that differences in vaccination levels were much greater at Category B and Category C schools than at Category A schools (Fig. 1).

## Discussion

Our study showed that only a small proportion of students were vaccinated against HBV, an expected finding since the vaccine was not part of India’s expanded program of immunisation prior to 2012. Most students surveyed had been born during the years 1997 to 2003, in a period when community awareness about HBV and its prevention through widespread vaccination was low [10, 20]. Further, the HBV vaccine available in India at that time was expensive as it was marketed primarily by one multi-national company [11].

Students in category A schools had a higher vaccination rate than those in category B and C schools. Also, the difference in vaccination rates among intervention and control schools was lowest in this category. This suggests that category A students, who probably belonged to a higher socioeconomic level, had higher levels of awareness. This could be from other sources such as family, internet, media and school, but the exact cause is outside the purview of this study. For Category B and C schools, the difference in vaccination between intervention groups and control group is much higher, suggesting that the efficacy and need for the intervention may both be higher in lower socioeconomic categories. The finding that students from category A schools had better rates of vaccination further supports the idea that awareness and affordability are the two main drivers of vaccination. These children came from better socioeconomic backgrounds, had more access to private health care, had more educated parents and had a greater capacity to afford the vaccine. In contrast, students at category B and C schools came from lower socioeconomic backgrounds, with poorer awareness levels and fewer resources at home.

Australian workers have studied HBV and HPV vaccination rates and shown that government sponsorship, affordability and culturally appropriate educational programs help make vaccination drives successful [22]. Our observations are in accordance with their findings. We found that in the absence of a government-sponsored program for HBV, the vaccination rates were low, and that a culturally appropriate awareness program through a movie set in India made a significant impact on children. We theorize that the falling price of the vaccine and its availability to students for free or nominal charges led to greater acceptance.

Periodic educational programs have been shown to improve knowledge and bring about behaviour change in both care givers [23] and students. We assessed HBV knowledge among students before and six weeks after a session of movie screening, the gap of 6 weeks was kept to assess sustainable awareness rather than immediate recall. School-based programs have helped risky behavioural patterns in adolescents [24]. We therefore worked on the principle that frequent educational programs through screening of a movie could improve awareness about HBV and bring about increased adoption of vaccination.

We observed that knowledge and awareness scores significantly improved after the movie was screened in all categories of schools. Further, the movie was a more interesting and engaging way of creating awareness than lectures. Even after a two-year follow-up period we observed an increase of more than double in the vaccination status of intervention schools (21% to 45%) in comparison to non-intervention schools whose vaccination status remained low (22%). The non-intervention schools’ vaccination status remained around the baseline vaccination status of interventional schools.

This study showed that the vaccination rate in category A schools was not different in intervention groups and control groups. Category A school children mostly belongs to higher socioeconomic and well educated families, so that vaccination rate was high in both group. The observation relates to the study done by Middleman et al. [25], where children with higher socioeconmic status had higher vaccination rate in comparison to children with lower socioecomic status.

In category B and C schools, the vaccination rate was higher in intervention groups as compared to non-intervention groups. Children of category B and C basically belonged to middle and low socioeconomic status. Their families were not so much educated about HBV disease and its prevention. Following the intervention, their basic knowledge increased about HBV prevention and a surge in vaccination occurred in these two categories. The implication is that lower and middle socioeconomic groups would benefit the most from an intervention of this nature.

There were several limitations of this study. First, between 10% and 15% of students were lost to follow-up due to school absences. Secondly results for the entire intervention group were not analysed at the two-year follow up. Thirdly, the school infrastructure and facilities were used as a proxy indicator for the socioeconomic background of students and their families. Fourthly, there could also be students who have changed schools or were absent between the two questionnaires. However we thought with large numbers this change will be acceptable. Fifthly, the movies were screenend in the school for children who may or may not further communicate, motivate and encourage there parents to get them vaccinated. Subsequently screening of parents or asking students whether they have discussed about the film with there parents will be a better method to assess school to community approach in future studies by adding another question in the second questionnaire. By this method we could have also assesed vacciantion level in the students who have shared the knowledge from video with their parents. Considering the diversity in the Indian population with regard to customs and traditions, the impact of these factors on vaccination rates was not considered. The customs, traditions and religious beliefs need to be looked at with respect to different demographic populations so as to implement methods which are appropriate and acceptable for the community. Lastly, other confounding factors such as general awareness due to advertisements and other programs were not considered and were beyond the scope of the study. Larger studies are needed in this field, as to build up this tool to implement programs in the community for various other causes.

## Conclusion

This study showed that school-based interventions can be used to enhance knowledge about HBV. There was an increase in awareness, knowledge and vaccination rates following the intervention. This tool is affordable and can help reach a larger audience. It can also be used for other causes relevant in the community. However larger studies and better assessment to validate the impact can help make this a robust method for various social causes.

## Abbreviations

HBsAg: Hepatitis B surface antigen
HBV: Hepatitis B virus
NGO: Non-Government organization
UP: Uttar Pradesh
WHO: World Health Organization
